# How evolutionary behavioural sciences can help us understand behaviour in a pandemic

**DOI:** 10.1093/emph/eoaa038

**Published:** 2020-10-24

**Authors:** Megan Arnot, Eva Brandl, O L K Campbell, Yuan Chen, Juan Du, Mark Dyble, Emily H Emmott, Erhao Ge, Luke D W Kretschmer, Ruth Mace, Alberto J C Micheletti, Sarah Nila, Sarah Peacey, Gul Deniz Salali, Hanzhi Zhang

**Affiliations:** e1 Department of Anthropology, University College London, 14 Taviton Street, London, UK; e2 State Key Laboratory of Grassland and Agro-ecosystems, School of Life Sciences, Lanzhou University, 222 Tianshui South Rd, Lanzhou, Gansu Province 730000, People's Republic of China; e3 Department of Epidemiology and Public Health, University College London, 1-19 Torrington Place, London, UK; e4 Institute for Advanced Study in Toulouse, Université Toulouse 1 Capitole, 1 esplanade de l’Université, 31080 Toulouse Cedex 06, France

**Keywords:** behavioural ecology, cultural evolution, COVID-19, lockdown, social distancing, behaviour change

## Abstract

The COVID-19 pandemic has brought science into the public eye and to the attention of governments more than ever before. Much of this attention is on work in epidemiology, virology and public health, with most behavioural advice in public health focusing squarely on ‘proximate’ determinants of behaviour. While epidemiological models are powerful tools to predict the spread of disease when human behaviour is stable, most do not incorporate behavioural change. The evolutionary basis of our preferences and the cultural evolutionary dynamics of our beliefs drive behavioural change, so understanding these evolutionary processes can help inform individual and government decision-making in the face of a pandemic.

**Lay summary:** The COVID-19 pandemic has brought behavioural sciences into the public eye: Without vaccinations, stopping the spread of the virus must rely on behaviour change by limiting contact between people. On the face of it, “stop seeing people” sounds simple. In practice, this is hard. Here we outline how an evolutionary perspective on behaviour change can provide additional insights. Evolutionary theory postulates that our psychology and behaviour did not evolve to maximize our health or that of others. Instead, individuals are expected to act to maximise their inclusive fitness (i.e, spreading our genes) – which can lead to a conflict between behaviours that are in the best interests for the individual, and behaviours that stop the spread of the virus. By examining the ultimate explanations of behaviour related to pandemic-management (such as behavioural compliance and social distancing), we conclude that “good of the group” arguments and “one size fits all” policies are unlikely to encourage behaviour change over the long-term. Sustained behaviour change to keep pandemics at bay is much more likely to emerge from environmental change, so governments and policy makers may need to facilitate significant social change – such as improving life experiences for disadvantaged groups.

## 1. INTRODUCTION

In theory, stopping the spread of viruses is simple: limit contact with other people and prevent transmission [1]. In practice, this is hard. While many individuals promptly respond to social distancing measures, others are resistant to change, and even do things that make matters worse. Scientists may advise governments to enforce behaviour in the absence of voluntary cooperation, e.g. through policing ‘lockdowns,’ as has happened in much of the world during the COVID-19 pandemic. Such policy decisions have potentially profound consequences for our survival, and also for our social and economic well-being. Therefore, we would benefit from using all the tools at our disposal to help governments and individuals make decisions successfully.

Understanding the fundamental principles underlying behaviour change may seem a bit of luxury in the maelstrom of a pandemic that has killed over a million people across the globe. Tried and tested public health policies learned through hard experience of managing other epidemics are certainly a priority in the early stages of any pandemic. Public health bodies try to inform the public of health risks, in the hope that will cause individuals to change their behaviour for their own good, and especially for the public good. However, evolutionary frameworks do provide some guiding principles behind human decision-making, which is absent from most of the models used to inform public health policy [2]. The focus in behavioural ecology is on how living things have evolved to respond in different ecological conditions [3]. There is a long tradition in behavioural ecology of examining how behaviour (of humans and other species) varies in response to demography, ecology and access to resources from an evolutionary perspective, which has relevance to understanding how we behave in a pandemic, so may help improve behavioural interventions. Everybody will change their behaviour in different ways according to their circumstances, but many of those differences are predictable in an evolutionary framework.

In evolutionary models of human behaviour, the currency determining the costs and benefits of behaviour is inclusive fitness. The reasons why evolution might favour such behaviours are often called the ‘ultimate’ explanation for behaviours [4]. The minutiae of decision-making cannot always be mapped directly onto fitness consequences, so currencies that may approximate to reproductive success are often used instead, both by behavioural ecologists modelling behaviour and presumably also by human brains when deciding how to behave. Evolutionary life history theory (Box 1) uses a cost−benefit scenario that makes explicit that the optimal behavioural responses of individuals depend on a range of contingencies; the most fundamental of these include age, sex, mortality risk in the environment and constraints.

Optimal decisions in terms of maximizing fitness involve trade-offs (Box 1). For example, as an individual living through a pandemic, we may have to decide whether to go out to work and risk infection in order to earn money for immediate and future needs, or to stay at home and avoid infection. Evolutionary models can theoretically unite different currencies, such as infection risk and economic benefit, through their impact on the common currency of reproductive success [5], or some proxy such as long-term survival. Such models highlight the need to take into account how avoiding disease increases chances of starvation or loss of livelihood, and how these jointly influence survival and reproductive success. Avoiding disease by social distancing reduces the likelihood of meeting a reproductive partner, and any associated loss of income/employment may hinder the opportunity to reproduce or invest in the well-being of your children or grandchildren—with these ‘costs’ potentially persisting into the future. Younger individuals of reproductive age therefore face different trade-offs from older individuals of post-reproductive age even before considering age-specific differences in mortality risk observed due to COVID-19 [6]. Infection risk is not the only consideration in optimal decision-making, and may not even be very significant in evolutionary terms.

When governments make policy decisions, the trade-offs are usually evaluated at the population level. Such decisions—like many of the most challenging global problems—are social dilemmas: there is a collective benefit from widespread cooperation across the population which the government wishes to foster, but individuals have an incentive to maximize their own personal welfare and ‘free ride’ on the cooperation of others. Whilst basic evolutionary and economic models of behaviour assume that self-interest is motivating for individuals, evolutionary models also reveal why individuals may opt to cooperate in line with the public good. There are plenty of theories as to why self-interest is also compatible with behaving cooperatively. Cooperation is most likely to evolve when it is based on kinship, reciprocity or reputational concerns (known as indirect reciprocity) (Box 2). These factors can only favour the evolution of cooperation in small or at best medium-sized groups [7]; it is difficult to keep track of defectors in larger groups, which may have implications for how governments design and implement policy based on voluntary cooperation.

Behavioural ecologists generally assume that in most cases our psychology is somehow equipped to evaluate inclusive fitness trade-offs through cues from our environment; our psychological preferences therefore guide us to behave in a broadly adaptive way. However, the assumption that fitness is maximized by our behaviour does not always hold. Evolution takes time to work, and full knowledge of what is happening may not be available. This is especially relevant when facing a new disease in a rapidly changing environment. Cultural transmission, which is an important evolutionary mechanism behind establishing our norms of behaviour, may not be as fast at spreading fitness-maximizing behaviour as the spread of the virus. It can also lead to the spread of behaviours that do not maximize inclusive fitness [8], nor benefit an individual in any wider sense (Box 3). Nor will it necessarily lead to behaviour that benefits the wider group; although some argue cultural transmission is better placed to generate the establishment of group-beneficial norms than is natural selection on genes [9].

A central insight from evolutionary theory is that our psychological preferences and behaviours evolved not to maximize our health, or the health of our group, but our inclusive fitness. Such insights can help us better understand why individual- and group-level behaviours may conflict with policies designed to mitigate the health and social impact of COVID-19. Here, we examine the underlying ‘ultimate’ causes of behaviour and decision-making and argue that it can help develop more effective strategies for tackling problems such as: compliance with health-promoting rules and social distancing, domestic violence, preventing the spread of misinformation and engendering cooperation within and between groups. While the topics covered below are by no means comprehensive, they provide examples of how an evolutionary approach can be used to understand the global challenges experienced during a pandemic.

## 2. COMPLIANCE

Long-term compliance with health guidelines, as required to contain COVID-19, requires rules that fit with our evolved preferences as much as is possible. Public health guidelines and advice are often predicated on the notion that knowledge of risk will improve compliance. Knowledge can be helpful, but lack of knowledge may not necessarily be the constraint on compliance. Individuals have different priorities based on their own circumstances and ecology, and behavioural change in response to exposure and knowledge of risk itself has its own complicated relationship with behavioural outcomes related to compliance [10].

To understand people’s response to competing risks, a life history framework is useful (Box 1). A central tenet of life history theory is that high mortality risk is associated with a preference for earlier and faster reproduction, or a ‘live fast die young’ strategy [11−14]. While the preference for reproduction over health is not necessarily verbalized, such a phenomenon has been observed in young African men who were less likely to respond to advice on wearing condoms if they were of a lower socioeconomic position, despite being the most at risk of HIV, because they were subject to a greater risk of mortality than wealthier individuals [15]. From a life history perspective, the reproductive benefits of unprotected sex (attracting partners, fathering offspring) outweighed the marginal benefits of reducing one of many mortality risks; lack of knowledge of HIV was not their reason for avoiding condom use [15]. Similarly, sex education does not necessarily reduce pregnancy rates among those teenagers with few opportunities to gain from continuing their education: They evaluate the costs of delaying reproduction as greater than the cost of leaving school [16]. As male reproductive success tends to be more variable than female reproductive success, risk-taking is generally considered to be more adaptive in males. This may underpin a greater prevalence in males of health-harming behaviours ranging from criminality [17] to a reluctance to wear face masks if they are perceived as ‘not cool’ [18].

**Figure 1.**
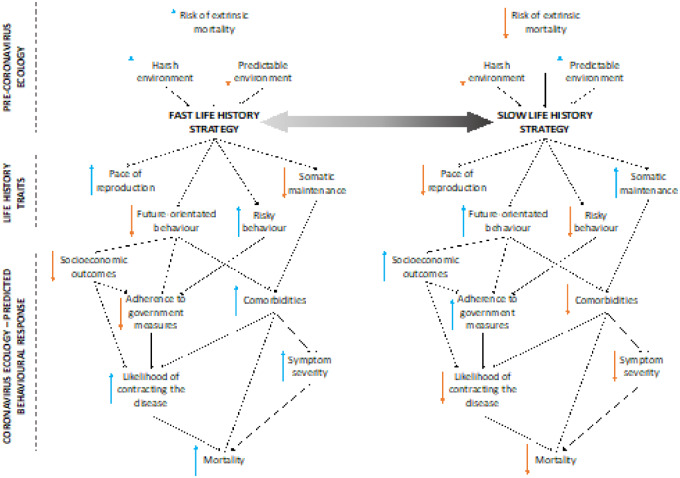
Visual description of the hypothetical relationship between ecological uncertainty, life history traits, the risk of contracting COVID-19, compliance and mortality. Upwards blue arrows indicate an increase in the trait, and downwards orange arrows a decrease, e.g. fast life history strategy is associated with an increased pace of reproduction

Figure 1 illustrates a range of ways in which a greater risk of extrinsic mortality might promote a faster life history strategy, resulting in more risky behaviour and a decreased likelihood of compliance. A fast life history strategy has been linked to poorer health outcomes (such as obesity [19]) and a lower socioeconomic position [20], meaning the individuals for whom the health risk is highest might be those least likely to respond to public health measures. Government lockdowns affect people of a lower socioeconomic position in a disproportionate way through creating greater economic insecurity [21], running the risk that an uncertain environment makes some people more likely to engage in risky behaviour and disobey government rules (Fig. 1). It is possible that the government and media strategies of constantly drawing attention to mortality resulting from the pandemic could have perverse effects through enhancing risk-taking, as the salience of mortality has been shown to cause people to prioritize speeding up reproduction, as recently observed in Indonesia in response to COVID-19 [22]. Alternatively, those with longer time-horizons may perceive the risk of COVID-19 as short-term and thus delay child-bearing until it is over, as recently predicted in the USA [23].

As our evolved instincts are often selfish or family oriented (Box 2), compliance with measures that benefit the public at some cost to ourselves rely on institutions enforcing punishment. Punishment could be enforced by the passage of laws or, more effectively just by reputational costs [24]. In the UK, whilst rules require individuals in certain categories that may be ill with or recently exposed to COVID-19 to quarantine for up to 14 days, any legal enforcement was extremely unlikely over the summer of 2020. A study found that, whilst the theoretical intention to quarantine if contacted by contact tracers was high (∼65%), <18% self-reported that they complied [25]. Non-adherence was associated with men, younger age groups, having a dependent child in the household, lower socio-economic status, greater hardship during the pandemic and working in a key sector.

Given that reputational costs are a powerful incentive to cooperate, but not easily enforced in larger populations, the support of local communities for enforcement of regulations needs to be strong. Despite our instincts to avoid punishment, individuals can prefer to live in environments where behaviour is controlled by institutional punishments (legal systems, police forces, governments, religions etc.) as we are willing to pay the costs (e.g. taxation, possible retribution) of institutions that enforce the public good if they are effective at improving the quality of life for everyone [26]. Acceptance of leadership may have evolved for precisely that reason [27]. If leaders are not effective, trust and the acceptance of social inequality necessary for leadership is likely to dissipate rapidly.

## 3. SOCIAL DISTANCING

A central policy for stopping the spread of COVID-19 is social distancing, which refers to measures that reduce the frequency and proximity of human-to-human contact, to reduce the rate of disease transmission in a community [28]. The most extreme version of such measures to contain COVID-19 involve ‘lockdowns’ which were first implemented in China, where Wuhan and its 11 million residents were placed under strict lockdown on 23 January 2020 [29]. This attempt at community containment included various social distancing measures such as school closures, the suspension of public transport and stay-at-home directives [30]. By March, similar measures were being implemented across the globe [31]. Although social distancing measures have been effective in reducing the rate of transmission of COVID-19, they have posed social challenges. Like most primates, humans are highly social animals [32]. We rely on social interactions within large and complex groups for cultural learning (Box 3) and support in raising children (Box 2) [33−35]. We have an evolutionary history of cooperative childrearing [36], meaning alloparental (i.e. non-parental) support is necessary for successful reproduction and childrearing. Alloparental investments have been associated with better child outcomes [37] and, in most populations, formal education via ‘institutional alloparenting’ is a key determinant of long-term wellbeing [38]. The disruption to learning as a result of a pandemic was observed during SARS, in which children experienced delayed developmental milestones such as counting and saying a complete sentence [39].

Constraining our social support networks by social distancing means that lower levels of practical support will be transferred between households. For parents, caring for children who would otherwise be at school or cared for by others can lead to increased stress, loss of income [40] and potentially delayed or reduced fertility [23]. This may have a profound effect for our most vulnerable children. Humans are one of the few primate species known to withdraw parental investment and, in extreme cases, commit infanticide where there is a lack of resources and alloparental support [41]. With social distancing severing support networks, some children may be put at increased risk of child neglect and abuse. School closures and holidays are associated with an increased incidence of child maltreatment [42]; and, in the UK, during the first month of lockdown there was a 1493% rise in the incidence of suspected abusive head trauma in infants [43].

Even without abuse, social distancing measures are likely to have a negative impact on children’s development. Evidence from non-pandemic circumstances shows that a long period of school absence is likely to lead to substantially reduced educational outcomes for children [44, 45]. Long period of isolation from peers is likely to be detrimental for socio-emotional development [46, 47]. For adolescents, teenagehood is a critical developmental period for socio-cultural learning [48, 49] reflected in their broadening of social networks with a greater focus on peers [48]. Constraining social ties through social distancing at this crucial developmental stage may be particularly detrimental [50]. Teenagers have been reported as more likely to flout social distancing rules, perhaps prioritizing mating effort and social opportunities over disease avoidance [51, 52]. While schools are hot-spots for influenza transmission [53], it is still less clear what role they play in the transmission of COVID-19 [54, 55]. However, there is evidence that they can act as spreaders, with this effect being particularly prevalent in secondary/high schools [56], thus potentially transmitting the virus to older individuals they interact with. As children and adolescents are physiologically less affected by COVID-19 than older members of the population [57], social distancing imposes a high cost to them for the benefit of adults [57].

Social distancing rules therefore pose dilemmas for families, as it is unclear whether the benefits of social distancing and avoiding the virus outweighs the immediate and long-term costs (e.g. potential fitness costs of higher mortality risk for infants or grandparents, lower mating opportunities for young adults, etc.). The magnitude of these costs may be missed by policy makers who, in Western contexts at least, typically view intensive parenting as the ‘normal’ form of childrearing and significantly underestimate the costs of severing a family’s social ties.

## 4. DOMESTIC VIOLENCE

Lockdown measures mean most people spend more time within their household than ever before, and under some circumstances, this is having harmful consequences. Domestic violence and femicide have increased during lockdown, which is generally attributed to the close confinement of victims and perpetrators and the removal of victims’ support systems, which both facilitate the violence [58]. An evolutionary approach suggests that a tendency to control a mate has a strategic function in a way that is distinct from a conventional analysis that views domestic violence as deviant behaviour in order to assert dominance [59]. If the tendency to resort to violence to control access to a mate has an evolved function, then it should have increased reproductive success in ancestral environments, through either securing more mates or more mating [60]. Intimate partner violence is indeed associated with higher marital fertility in a forager-horticulturalist population [61], where men may use wife abuse both as a means of increasing family size, and also as a means of pursuing their own extra-marital affairs [62]. Domestic abuse may also be coercive behaviour used to ensure continued access to a sexual partner. Indeed, survey data collected by Safe Lives [63] during the pandemic shows that while a large proportion of abusers are current partners (∼20%), a greater proportion (∼63%) are ex-partners.

Under lockdown, we could be witnessing increased attempts by ex-partners to regain control and coerce women into re-entering into a partnership with them. Safe Lives [63] argues that the uncertainty of the current period may cause victims to return to their perpetrators, and abusers may recognize this and use it to their advantage. Risk of domestic violence does decline as women age, with younger women being more likely to be a victim due to their higher fecundity and mate value (i.e. increasing the fitness benefit of coercion for the abuser) [64]. This pattern was seen following Hurricane Katrina, where it was observed that younger women were more likely to experience an increase in violence [65]. Furthermore, financial insecurity is associated with unstable partnerships [66]; the loss of jobs caused by pandemic mitigation strategies may increase women’s incentives to separate. This may motivate some males to retain partners through coercion, while at the same time lockdowns can inhibit female escape strategies and reduce their bargaining power, thus increasing instances of domestic violence.

There is an understanding that the rise in abuse is not caused by new perpetrators but by previous abusers whose violence has increased [67]. Given the financial instability of the post-COVID world, new abusers may have emerged or will emerge, and data should be collected to elucidate this. We cannot assume that this rise in domestic violence will decrease as lockdowns end and women’s refuge services resume, as previous research into abuse following natural disasters has shown that increased demands for services persists for up to a year following the incident [68].

We suggest that policy should focus on demographic groups that an evolutionary approach would highlight as being at a heightened risk of abuse, such as younger women and women whose partners are under economic stress or risk of job loss. Additionally, policies that reduce the bargaining power of women should be highlighted as facilitating domestic violence. For example, in the UK, the aggregation of child benefits, which had previously gone to the mother, into universal credit which is allocated to the head of the household (usually the man) removes a crucial lifeline to victims of domestic violence, making them further reliant on their abusers [69]. Using evolutionary theory to understand under what circumstances abuse might be expected may allow policy makers to target certain individuals and anticipate when during the pandemic violence may increase.

## 5. CONTACT TRACING

Our behaviour is determined partly by our ecology, and also partly determined by culture, i.e. local conventions, institutions and symbolic practices that exist upon a common recognition and acceptance by all group members. Culture also evolves over time (Box 3). How people respond to government guidelines may be influenced strongly by the people they are surrounded by and the culture they are a part of Ref. [10]. In Western, Educated, Industrialized, Rich and Democratic (WEIRD) societies [70], there is a rising scepticism and resistance against contact-tracing measures, which results from perceiving privacy as a moral imperative, safeguarding ideals of political freedom and moral autonomy [71]. A strong belief in individual rights to privacy hampers the introduction of surveillance and contact-tracing infrastructures as these measures are politically costly [72]. The strength of this belief is culturally contingent [73]; in some East Asian countries mass surveillance has been the norm for some time. This means that there is greater acceptance of various contact tracing technologies, and the existing tracking infrastructure has given a head start to the epidemic response [74]. Increased acceptance of contract-tracing in East Asian countries may also stem from experience with recent lethal epidemic outbreaks such as SARS and MERS [75].

Some argue that such culturally specific attitudes towards individual vs collective welfare stem from historical differences in farming practices [76]. Experiments show people from WEIRD populations are more likely to adopt more asocial, individualistic learning strategies than other populations [77, 78]. Many attributed national differences in epidemic response to cultural differences of the collectivist attitude, that is, the tendency to sacrifice personal interests for collective gains, in contrast to ‘individualism’, which prioritizes individual autonomy [79]. Some attempted to draw a causal link between collectivism and historical exposure to pathogen stress experienced by the group [78], but this correlation does not hold after differences in government effectiveness is taken into consideration [80]. The reality is likely more complex. Immediate concerns for material insecurity, historical contingencies, such as the spread of Protestant values of self-reliance and individualism [81], and reputational concerns are all likely to shape the level of collectivism in different cultural groups [80].

One way to tackle non-compliance to contact tracing is to obtain a consensus from the public that we are now in a different social context from pre-pandemic times, so a new moral norm (i.e. more neutral attitudes towards personal data disclosure for contact tracing) is required in the new ‘ongoing pandemic’ context. Effective contact tracing through mass surveillance—as seen in South Korea—in some instances curbs the individual right to privacy. But a realistic understanding of the epidemic threat would change the context of our normative conventions and facilitate behavioural changes away from norms about privacy that may no longer be appropriate in a pandemic. In the USA, mandatory HIV screening for pregnant women was considered justifiable, as benefits outweighed the costs of the privacy breach [72]. Contrary to what many feared, establishing a new norm of sharing personal information to aid effective contact-tracing during a pandemic does not necessarily jeopardize the long-term moral norms regarding privacy protection, as social norms in human societies are often context specific [82] and we would eventually return to a ‘pandemic-free’ social context. Understanding which cultures would be more receptive to different technologies may help governments market them to the public.

## 6. MISINFORMATION, CONSPIRACY THEORIES AND VACCINE UPTAKE

During a pandemic, social learning strategies, which have been adaptive in our evolutionary past, may be harmful (and maladaptive) under new conditions [83]. For example, conformist bias acting inside online ‘echo chambers’ and prestige bias may result in the spread of misinformation [84, 85] (see Box 3 for definitions). Scientific controversies over wearing face masks [86] means that—in the absence of government implementation—usage does not take off until a sufficient number of people in the community start wearing them, so that it becomes a cultural norm. The COVID-19 epidemic has produced ‘fake news’, conspiracy theories, and dubious ‘alternative’ remedies purported to prevent or cure the virus. Many people in the UK and the USA hold such ideas [87, 88], which often proliferate in pandemics [89, 90].

Misinformation and conspiracy theories are clearly a barrier to curbing the spread of the virus. The development and implementation of a vaccine is one of the most promising ways of eradicating COVID-19; however, for this to work, enough people have to be receptive to the idea of being vaccinated. Vaccines are central in many conspiracy theories, with conspiracy beliefs often attributing unseen causes of important events to a powerful coalition secretly working to cause harm [91]. When the error costs of not perceiving a threat are potentially more catastrophic than its over-detection, selection favours a bias towards over-detection [92, 93], meaning people adopt vaccine-avoidant behaviours. ‘Anti-vaxxers’ believe that governments and pharmaceutical companies are covering up information for their own gain. For example, many believe that due to the possible profits to be made by pharmaceutical companies, they are covering up negative side-effects or over-stating the efficacy of vaccines [94]. Clusters of people who hold anti-vaccination beliefs can become entangled with undecided individuals and influence them [95]. Rather than intentionally causing harm to the public, believers perceive themselves as participating in a common cause [96] and form ‘echo chambers’, in which they only encounter perspectives that reinforce their own. The spread of anti-vaccine sentiment and the disease itself may co-evolve [97]—we may not hear much from anti-vaccine campaigners when an epidemic is at its height, but as the disease disappears anti-vaccine sentiment can help to build up the next wave of infection.

Vaccination decisions are also shaped by omission bias—when people are faced with a choice between taking a specific action or doing nothing, they often prefer to do nothing if taking action introduces costs or risks that would not have impacted them otherwise [98]. Even when the risk of catching a disease is higher than that of vaccine side-effects, people prefer not to vaccinate [98−100]. Similarly, our sense of disgust may play a role in vaccine hesitancy [100], which is associated with aversion to blood and needles [101] and concern for bodily purity [102, 103]. This is evident in the misconception that vaccines contain harmful ‘toxins’ [94, 104]. Superstitious treatments may proliferate when people observe and copy others using them [105]. Determining what cures disease is difficult when patients can recover spontaneously. This is particularly relevant with COVID-19, as many people are able to recover at home without specialist treatment [106]. Ineffective remedies may be popular because their very ineffectiveness means patients are ill for longer, prolonging usage, which is then copied more frequently [105].

However, the same processes that produce misinformation can motivate compliance with effective measures. As people are more likely to trust information and conform to behaviours they observe in their in-group, appeals by peers are more successful. Shelby and Ernst [96] recommend parents whose un-vaccinated children contracted preventable diseases and parents who immunized their children without adverse effects should share their stories. If people are told that many peers vaccinate, they are more likely to follow suit [107]. Engaging social media users to combat misinformation from others in their network has proven effective in previous outbreaks. For example, a conspiracy theory circulated stating the Zika virus was being transmitted using genetically modified mosquitoes. This was successfully countered on social media by providing links to corrective information and encouraging other users to refute misinformation [108]. Similar methods are now being implemented globally, with the track-and-trace system and mask wearing being promoted in the UK through government paid advertisements by ex-*Love Island* contestants and other influencers [109]. Ethnographic studies can help shed light on local views and responses to an outbreak. Standard bio-medical messages, such as ‘science and medicine are our only hope’, do not lead to behaviour change [110], in part because communities often have different beliefs on the effectiveness of treatment methods for different conditions. For example, while Congo hunter-gatherers trust in the effectiveness of Western medicine for certain diseases, for others they rely on traditional practices [111]. Anthropological studies on the local perceptions of the Ebola outbreak found that while certain cultural practices contributed to the spread of the disease, others can be used to slow down epidemics especially considering that most of these communities already lived in high mortality environments [112]. Identifying health-enhancing cultural practices and incorporating them in the design of public health messaging can be helpful.

If a practice contributes to group identity, many people will only abandon it if the link between that behaviour and their group identity can be disrupted [113]. For example, campaigns to end female genital mutilation (FGM) can produce a backlash if they imply that local values must be abandoned or supplanted by outside ones [114]. Doing so threatens the target audience’s identity. Interventions were more successful if they employ locals to model anti-FGM views and emphasize that conflicting attitudes already exist within populations that practice FGM [114]. These interventions show that people who share the target audience’s cultural values can reject FGM, and that doing so is compatible with being a member of that group.

Policymakers should be aware that cultural groups, and those within groups, may have different beliefs about the effectiveness of treatment methods and vaccines against COVID-19. It is important to first understand these beliefs and co-design health promotion messages with local groups [115]. Stigmatization risks entrenching hostile attitudes further [113]. Campaigns are likely to be more successful if they rely on peer interactions with people that members of the target audience share a social identity with, e.g. by encouraging people who already follow guidelines to become peer educators in their real-life social networks. Peer intervention (e.g. on social media) can be used to refute harmful information that is liable to prevent people from following guidelines, such as the idea that COVID-19 is caused by 5G masts [108]. Conformist social influence can be used to emphasize how others in a social environment or target audience peer group are currently complying with regulations so that others then adopt these behaviours [113].

In the absence of a vaccine, the primary tool at our disposal to prevent the spread of the virus is behavioural change, such as mask wearing and social distancing. Misinformation and conspiracy theories are found to be one of the primary reasons that people are hesitant to adopt these behaviours [116]. Cultural evolutionary theory can help us understand who is most vulnerable in regard to believing untruths about factors relating to COVID-19, and what we can do to minimize the spread of such misinformation.

## 7. INTERNATIONAL COOPERATION

We respond to crises not only just as individuals, but also as members of a series of nested communities. Often we have to entrust institutions with devising and enforcing health-related policies on behalf of the whole group. Modern states have multiple levels of organization (national, regional, municipal), and the authority granted to each of them varies greatly depending on the government system [117]. Supranational bodies, such as the European Union or the World Health Organization, play increasingly important roles in guiding policy or coordinating international initiatives on preventative measures [118]. Therefore, a successful response to the COVID-19 pandemic requires not only cooperation between individuals, but also intercommunity coordination, both between nations (international cooperation) and between levels of governance within a nation (intergovernmental cooperation).

Evolutionary behavioural sciences are being applied to investigating the evolution of societal organization and the drivers of intergroup cooperation, employing historical data analysis, experimental studies, and mathematical modelling. Mathematical models have started to explore how our species’ ability to form coalitions has made these shifts possible, exploring drivers of cooperation especially in the context of warfare [119, 120]. Throughout human evolution, cooperation between groups has been driven by two main classes of benefits: protection from common threats and resource sharing, especially during times of shortfall (‘risk pooling’) or if some resources are not available locally [121]. Both these benefits are relevant in the context of the current pandemic, as COVID-19 is a threat to all countries and many communities have faced shortages of medical equipment that have been mitigated, at least in part, through international and intergovernmental cooperation [122].

Despite the potential benefits, collaboration between communities can often fail; just like individuals, groups might experience different costs and benefits that can result in conflicts of interest and, crucially, these may depend on the ‘ecology’ experienced by different groups and their current status [121]. For example, a recent evolutionary game theoretic model predicts that resource inequalities between players can facilitate intergroup cooperation, because the rich invest more in the public good to protect their wealth and this creates the conditions for the poor to contribute as well [123]. However, a behavioural experiment simulating individuals ability to mitigate climate change has demonstrated that, when resource inequalities are coupled with higher risks for poorer groups, conflict can ensue, as richer players are both at less risk and less incentivized to invest [124]. But to understand which conditions will result in intergroup cooperation, it is not enough to consider potential conflicts between groups only at one level. The interests of groups at multiple organizational levels—and ultimately of the individual citizens within them—must be analysed simultaneously, since conflict within lower levels might influence cooperation between higher levels [120, 121,125].

An evolutionary approach suggests reasons behind suboptimal responses to the COVID-19 pandemic from states and communities are likely to be found in these conflicts of interest and their ‘ecological’ drivers. It is possible that South Korea and Taiwan responded more effectively than Italy or the USA because the former implemented unified national plans rapidly, while the latter struggled, having multiple decision-making centres in regions/states [126]. Although greater centralization is not necessarily the only possible solution. Research in evolutionary anthropology has shown that conflict resolution is one of the primary functions of leaders in both in small-scale and large-scale human societies, whether egalitarian or stratified [127]. A central leadership capable of mediating between regions, together with clarification of national and regional roles, might be the key to a more effective response, especially in federal systems as an initially successful response in Germany suggests [128]. A recent mathematical model of the evolution of military alliances [120] suggests that it might be necessary for conflicts between lower levels (e.g. cities, regions) to be resolved or kept in check for cooperation between higher levels (e.g. states) to be sustained. Given how easily the virus is spread, countries working together to share vaccine developments, including subsidizing those countries that cannot afford it, is likely to be essential to eradicating this disease globally. 

## 8. CONCLUSIONS

Evolutionary insights help explain the underlying drivers of behaviour, which can help explain why some people take more risks and may not comply with government rules, and why conflicts of interest between generations, between partners or between groups can all impede pandemic mitigation strategies. While attention to conflicts of interest is not exclusive to evolutionary theory, the insights from evolutionary approaches to behaviour can inspire novel solutions, complimenting work in political science, economics and public health on national- and international-level responses to the current pandemic [129] as evidenced in previous responses, such as with Ebola [130]. An evolutionary framework gives guidance as to what is likely to be sustainable in terms of policy to mitigate the costs of this disease. Three main guiding conclusions are following:

- ‘Good of the group’ arguments will not go far.
  Whilst individuals are willing to pay costs for the good of society, anything that involves long-term costs to the individual may not be sustainable unless balanced by other motivations to cooperate. Individual and family-based incentives need to be prioritized. Reputational costs are effective at the local level, but are highly context specific and may vary between communities. Top down diktats will be judged on their success at improving the lives of individuals. Anything that is generating conflict in society, from elections to (trade) wars, is likely to make large-scale cooperation at a national or international level more difficult to achieve.
- Behaviour is heterogenous.
  ‘One size fits all’, while improving clarity of message, does not acknowledge the very different costs and benefits experienced by different individuals in society, which will lead to non-compliance. Social distancing policies may need to make exceptions for different kinds of interactions (such as forming ‘social bubbles’ with elderly relatives or romantic partners living alone). Otherwise regulations are undermined by too many rule breakers—including public figures [131]—which can lead to the breakdown of general compliance. Similarly, across societies, heterogeneity in social conditions, ecological context and mortality hazard will mean that similar policies are met with different reactions in different communities. Different rates of infection in different groups may be due to behavioural rather than physiological differences, and people of different sociodemographic status may behave differently when faced with options including disease risk, due to different costs and benefits. Disease history is influenced by past behaviours and thus pre-existing conditions can differ between groups for behavioural reasons. Experience of current or historical oppression [132, 133] will play a role. Many of the harmful effects of risky environments will fall on the same individuals and thus exacerbate existing social, economic and health inequalities; e.g. those from ethnic minorities, those of a lower socioeconomic position, those who are not represented by their leaders, and those without obvious pathways to wealth and status, are all among those that may prioritize behaviours that do not protect them from COVID-19, and thus increase their own risk of infection. The interaction of social, socioeconomic, and biological factors could easily drive some of the unexplained socioeconomic and ethnic patterns of infection with COVID-19 [6].
- Behaviour change is linked to a change in ecology.
  A behavioural ecology perspective highlights that sustained behaviour change is much more likely to emerge from environmental changes, rather than by just telling people how to behave. The widespread adoption of long-term changes in behaviour that would help keep pandemics at bay may require profound ecological and structural changes that improve life experiences, particularly for disadvantaged groups. Policies may need to look at modifying the costs and benefits of certain lifestyles or behaviours in favour of more security and prosperity. This involves not only just modifying the risks of dangerous jobs in cleaning, nursing or public transportation, but also improving neighbourhoods, general public health and general education and reducing other impediments to security such as racism. Improving the prospects of an individual by changing their socioeconomic and physical environment is clearly much more challenging and costly for governments than just issuing advice about behaviour. But behavioural ecology, and other evolutionary frameworks, suggest there are few short cuts to successful mitigation strategies.

## AUTHORS’ CONTRIBUTIONS

All authors contributed for manuscript writing and paper editing. 

## GLOSSARY

**Adaptive.** A fixed or flexible behavioural response that is optimal; it maximizes the inclusive fitness of an individual in a particular ecology.

**Behavioural ecology.** The study of the evolutionary basis of behaviour as an adaptation to local ecological conditions.

**Cultural transmission.** Transmission of cultural traits (i.e. beliefs, skills, ideas, etc.) between individuals or within or between groups.

**Currency.** The quantity that is being maximized by individuals or by a process.

**(Darwinian) Fitness.** Genetic contribution of an individual to the next generation, relative to the average for the population.

**Inclusive fitness.** A generalization of Darwinian fitness that accounts for the fitness of related individuals who share genes by direct descent. Conceptualized as the sum of an individual’s reproduction and that of others, weighted by their genetic relatedness (r).

**Norms.** Shared rules and expectations that govern the behaviour of people within specific social and cultural groups.

**Reciprocity.** Performance of a cooperative act in which one agent provides a benefit to another on the premise that the other shall return the favour in due course. Any short-term cost associated with such an act is counterbalanced by the benefit obtained over repeated interactions.

**Indirect reciprocity.** Performance of a cooperative act where reciprocation does not come necessarily from the receiver but from the wider community as a reward for status or reputation as a co-operator.

**Reproductive success.** A measure of the number of viable offspring who in turn go on to have viable offspring.

Box 1 Life history theory Evolutionary life history theory predicts the optimal timing of key life events (e.g. growth, reproduction, death). Initially developed to understand the phenotypic variation in animal behaviour and morphology, life history theory is based on the idea that resources, such as time and energy, are finite [134]. Individuals face ‘trade-offs’ regarding how to optimally allocate resources, with phenotypes that allocate resources to maximize Darwinian fitness being selected for [135, 136]. Optimal resource allocation is contingent upon the risk of extrinsic mortality (risk of death from an external force, such as a disease) [136] and degree of ecological uncertainty/harshness [12]. More energy is allocated to reproduction when the threat of mortality is greater, whereas when the risk of mortality is lower, energy can be invested into somatic activities and more parental investment. One classic trade-off studied from within a life history framework is the timing of first reproduction, the point at which an individual forgoes investment in growth in favour of increasing direct fitness [137]. Harsher environments tend to associate with an earlier age of first birth [138], greater number of offspring [139], an increased rate of senescence [140], risky behaviour [13] and less future orientated thinking [12]. Thus, ecological uncertainty is associated with behaviours that are more rewarding in the short-term. Behavioural variation emerges with age [141], sex [142] and other population characteristics [143]. From an evolutionary perspective, post-reproductive individuals can only increase their inclusive fitness by investing in their kin, and indeed menopause may have evolved precisely to enable this [144]. This is why intergenerational support within families tends to flow down generations [145], even if governments are more inclined to spend money gathered from taxing the younger generations on the older generation [146]. As the presence of a virus imposes a new risk of extrinsic mortality, life history theory lends itself as an appropriate framework to understand variation in behavioural responses to the risk of catching COVID-19 and to compliance with emergency regulations. It should be noted that the application of life history theory to humans has drawn recent criticism due to the way in which the theory was abstracted from animal biology into human behavioural ecology and evolutionary psychology [147, 148]. For example, life history theory was developed originally for comparison across taxa rather than between individuals of the same species [147]. Furthermore, predictions are so context-specific that broad generalizations may be hard to establish. It is currently unclear whether humans follow a coherent life history strategy, or whether ‘traditional’ human life history predictors (e.g. life expectancy) are always associated in the predicted direction with life history traits, such as fertility and mortality [149]. Despite this, the logic of trade-offs and optimality modelling of life history is still useful for understanding human behaviour and decision-making under the conditions of a global pandemic.

Box 2 Cooperation A behaviour performed by an individual (the ‘actor’) is cooperative if it results in a fitness benefit for other individuals (the ‘recipients’). Natural selection favours cooperation if the actor obtains a fitness benefit either for genetically related individuals (indirect fitness benefits, where benefits are weighted by degree of relatedness) or for themselves (direct fitness benefits) [150, 151]. Inclusive fitness theory predicts that these indirect benefits drive cooperation within families [150]. However, it also highlights that individuals have different fitness optima, so there can be conflicts of interest between males and females, between parents and their children, and between siblings competing over parental resources [152, 153]. Conflict can decrease when some individuals are no longer reproductive, as exemplified by grandmothers participating in childcare responsibilities [154]. Direct benefits explanations for cooperation rely on reciprocity, reputation or enforcement. While potentially powerful, direct reciprocity—i.e. repeated cooperation between the same two individuals—relates to behaviour in dyads, not the wider population. Indirect reciprocity, where reciprocation is expected not from the beneficiary but from other community members, can sustain cooperation in larger group sizes; it is sustained by mechanisms such as gossip that can promote an individual’s reputation for being a co-operator (though such mechanisms become a lot harder in very large groups) [155]. Behavioural economics research suggests that reputation effects can help solve social dilemmas [155] and, in some small-scale societies, a higher reputation is associated with additional benefits that can translate into fitness, such as greater social support [156]. Finally, cooperation can be maintained by enforcement mechanisms—including punishment, rewards, sanctioning and policing [151]; this is especially important in very large populations, where reputation effects attenuate [157]. How to encourage individuals to pay personal costs for the public good is a key issue in a pandemic [158]. Local community support for governmental enforcement of rules is important given that reputational costs and benefits are likely to be key.

Box 3 Cultural evolution Cultural evolution is a field of research that suggests that cultural traits (e.g. ideas, skills, artefacts, etc.), like biological ones, are transmitted from person to person, exhibit variation, and are subject to selection [159]. These traits can be studied quantitatively using an evolutionary framework. Human culture is cumulative: our knowledge and skills accumulate over generations and increase in diversity and complexity [160].
**Biased learning in cultural evolution.** Biases in whom we learn from have evolved because individuals who have those biases were better able to survive and reproduce [8], especially when the costs and benefits of behaviour are hard for an individual to estimate. For example, prestige bias (copying/learning from highly respected and admired individuals in a group) can be adaptive if prestige is associated with high skill and knowledge [161]. Conformist bias (copying what the majority does or believes in), can be adaptive unless environments change too rapidly or individual learning is error free [8, 162]. By facilitating the spread of specific norms and behaviours, evolved learning mechanisms also contribute to the formation of cultural groups with shared beliefs, social norms and symbolic markers [163, 164], which can be linked to in-group favouritism and cooperation beyond the family [165].
**Evolution of norms and social institutions.** Humans are exceptional collective thinkers. By the age of three, human children already recognize mutually binding commitments to joint goals [166], and have sophisticated understanding of the context-specificity of normative rules in their pretend games [82]. In small-scaled hunter-gatherer societies, social conventions exist that help enforce an egalitarian social structure [167, 168]. In large-scale societies, cooperative rules are often realized and enforced through institutions (governments, police, religions), which—in the past 13 000 years—have increased in scope and complexity [169, 170].
